# Single-walled carbon nanotube-mediated plasmid DNA delivery in the model monocot *Brachypodium distachyon*

**DOI:** 10.55730/1300-0152.2796

**Published:** 2026-01-04

**Authors:** Kaya İŞLEYEN BİLGE, Bahar SOĞUTMAZ ÖZDEMİR

**Affiliations:** Department of Genetics and Bioengineering, Faculty of Engineering and Natural Sciences, Yeditepe University, İstanbul, Turkiye

**Keywords:** Single-walled carbon nanotubes (SWCNTs), *Brachypodium distachyon*, *b-glucuronidase* (GUS) gene, transient gene expression, callus-based assays, in-planta gene delivery

## Abstract

**Background/aim:**

Plant genetic engineering is a valuable approach for improving stress tolerance, crop productivity, and quality traits. However, its progress is limited by the drawbacks of traditional transformation methods. Commonly used techniques, such as *Agrobacterium*-mediated transformation and biolistics, are restricted by genotype dependency, lengthy tissue culture steps, and low efficiency, particularly in monocot species. Therefore, the development of alternative gene delivery strategies remains an ongoing research focus in plant biotechnology. Nanoparticle-based delivery systems have recently gained attention due to their physiochemical properties, biocompatibility, and potential for alternative gene delivery approaches in plant systems.

**Materials and methods:**

We investigated the use of single-walled carbon nanotubes (SWCNTs) as nanocarriers for plasmid DNA delivery in the model monocot plant *Brachypodium distachyon*. SWCNTs were functionalized with polyethyleneimine (PEI) and plasmid DNA (pCAMBIA1301) was loaded via electrografting. Successful complex formation was confirmed with transmission electron microscopy (TEM), atomic force microscopy (AFM), and zeta potential measurements.

**Results:**

The TEM and AFM analyses revealed that the SWCNTs retained a thin, elongated needle-like structure, consistent with their ability to pass through the plant cell wall. Zeta potential measurements showed a negative surface charge of −43 mV for COOH–SWCNTs that shifted to +56 mV after PEI functionalization, enabling electrostatic binding of plasmid DNA. pDNA–SWCNT complexes at different mass ratios were applied to both callus and seed explants to evaluate their delivery potential. Successful delivery was confirmed by *b-glucuronidase* (GUS) reporter gene expression in both callus tissues and seeds, with ImageJ-based quantification showing that the 3:1 SWCNT:pDNA ratio yielded the highest mean signal intensity.

**Conclusion:**

Our findings show that the SWCNT-based system enabled the delivery of a large plasmid (~12 kb), supporting transient reporter gene expression in monocot tissues. Overall, the data suggest that SWCNT-mediated delivery represents a promising technique for DNA delivery in monocots, and further optimization is expected to improve its efficiency.

## 1. Introduction

Advances in genetic engineering have both facilitated the functional analysis of plant biological mechanisms and enabled enhancement of agriculturally important traits. Especially with development of new genome editing tools like clustered regularly interspaced short palindromic repeat (CRISPR)-based systems, numerous studies have focused on improving crop yield, stress tolerance, and disease resistance. However, plant transformation remains a major bottleneck in plant biotechnology, and the search for alternative, efficient, and species-independent transformation methods is ongoing (Keshavareddy et al., 2018). The well-established plant transformation methods, such as *Agrobacterium*-mediated transformation and biolistics, are widely used but have significant limitations (Table). For *Agrobacterium*-mediated delivery, these include restricted host range, low efficiency in monocots, bacterial contamination leading to necrosis, and insertional mutagenesis (Gelvin, 2017). On the other hand, biolistics can be applied to any species, but often causes physical tissue damage and random transgene integration with multiple copies. Furthermore, most of these conventional methods rely on tissue culture and regeneration, which is labor intensive, time consuming, and dependent on genotype, creating a challenge for the transformation of important crops. To overcome these limitations, in planta transformation strategies that bypass tissue culture by introducing DNA directly into intact plant tissues have gained attention. Several in planta approaches have been developed such as pollen tube pathway-mediated transformation, floral dipping, floral spray, meristem-targeted transformation systems, silicon carbide whiskers, and seed imbibition methods; however, their efficiencies are generally low and they are often limited to specific plant species (Luo and Wu, 1988; Clough and Bent, 1998; Chung et al., 2000; Xiao et al., 2023).

Recently, nanoparticle (NP)-mediated delivery systems have emerged as a promising approach for plant gene delivery due to their unique physiochemical properties, biocompatibility, and species-independent application without genome integration (Demirer et al., 2019a, Kwak et al., 2019; Lowry et al., 2024). Compared with their extensive use in mammalian systems, the application of NPs in plants has progressed more slowly, largely due to the plant cell wall, which acts as a physical barrier to delivery. Although NP-mediated delivery has been explored in both dicot and monocot species, early optimization and proof of concept studies have mostly focused on dicot model plants such as *Nicotiana benthamiana* or *Arabidopsis thaliana*, mainly because these systems are well established with high regeneration rates, and are also suited for transient expression assays (Cunningham et al., 2018). In addition, differences in tissue anatomy between dicots and monocots can affect NP uptake and movement within plants, potentially posing additional barriers in monocot tissues (Vejlupkova et al., 2020; Zhang et al., 2023). As a result, NP-mediated systems have not been fully explored for functional genomic studies in plants, particularly in crop species with limited transformation efficiency (She et al., 2025). Therefore, developing an efficient and practical NP-based DNA delivery strategy for model monocots such as *Brachypodium distachyon* would significantly accelerate functional genomics studies and biotechnological applications for agricultural improvement.

The main advantages of NPs that have gained attention are their adjustable physiochemical characteristics such as size, shape, and surface charge, which can be engineered according to the purpose. One of the first demonstrations of NP-mediated delivery in plants was conducted by Torney et al. (2007), who used gold capped mesoporous silica NPs (MSNs) via biolistic delivery. Since then, various nanomaterials including single-walled carbon nanotubes (SWCNTs), multiwalled carbon nanotubes (MWCNTs), DNA nanostructures, clay nanosheets, calcium phosphate NPs, carbon dots, and selenium NPs have been successfully used for DNA, RNA, and protein delivery into plant cells (Liu et al, 2009; Wang et al., 2010; Serag et al., 2011; Naqvi et al., 2012; Mitter et al., 2017; Zhang et al., 2019; Şener et al., 2026). Different delivery mechanisms such as biolistics, sonoporation, and magnetic field-assisted transport have been explored.

Among these NPs, carbon nanotubes (CNTs) stand out due to their high aspect ratio, mechanical strength, and ability to penetrate plant cell walls without the need for external mechanical force (Demirer et al 2019a; Kwak et al., 2019). CNTs consist of cylindrical graphene tubes typically 1–2 nm in diameter for SWCNTs, while MWCNTs consist of interlinked nanotubes with diameters reaching up to 100 nm. Studies using MWCNTs show that their delivery efficiency is lower, likely due to their larger size, which limits their passage from the plant cell wall (Karousis et al., 2010). In contrast, SWCNTs have demonstrated a higher delivery efficiency by passing through the cell walls of intact plant cells from different species, including *Nicotiana tabacum*, *Oryza sativa*, *Triticum aestivum*, *Erica sativa*, and *Arabidopsis thaliana*, indicating that this method is species-independent (Burlaka et al., 2015; Demirer et al., 2019a, 2019b; Kwak et al., 2019; Dunbar et al., 2022; Pinyokham et al., 2022; Şener et al., 2025). Previous studies have shown that SWCNTs are capable of delivering DNA, siRNA, and proteins as cargo into intact plant cells and protoplasts (Demirer et al., 2019a, 2020). Surface modification of SWCNTs has been shown to increase DNA binding by promoting electrostatic interactions and effective interaction with plants cells. Polyethylenimine (PEI) is one of the most widely used functionalization agents and has been reported to support plasmid delivery in plants (Demirer et al, 2019a; Ali et al., 2022; Dunbar et al., 2022; Santana et al., 2022; Şener et al., 2026). Furthermore, alternative functionalization strategies have been reported for organelle-specific targeting including chitosan-modified SWCNTs, which improved cellular uptake and demonstrated targeted delivery to chloroplasts (Kwak et al., 2019), while another study employed peptide functionalized SWCNTs to deliver plasmid DNA to mitochondria (Law et al., 2024). Together these studies highlight that surface functionalization is a critical element of both delivery efficiency and subcellular targeting in plant cells.

Transient expression without genome integration has been observed with reporter genes such as GFP, GUS, and mCherry, with reporter gene expression declining postinfiltration, consistent with nonintegrative delivery mechanisms (Demirer et al., 2019a; Dunbar et al., 2022; Pinyokham et al., 2022). Dunbar et al. (2022) stated that reporter gene-based optimization studies demonstrated that SWCNTs can penetrate plant tissues, including the seed coat of rice, supporting their potential as delivery vehicles. In the same study, SWCNT-mediated delivery of CRISPR/Cas9 plasmids into rice seeds resulted in preliminary genome editing events at low frequency, highlighting the feasibility of this approach with further optimization. The nonintegrative nature of SWCNT-based delivery systems makes them particularly attractive for genome editing applications in which transient expression of CRISPR/Cas components could minimize regulatory concerns with genetically modified organisms.

Despite recent progress, NP-mediated gene delivery in monocot plants remains limited, and studies using SWCNTs in in vitro callus tissue and in planta seed-based delivery approaches are still scarce (Burlaka et al., 2015; Dunbar et al., 2022; Pinyokham et al., 2022). Considering the limitations of the traditional transformation techniques, the utilization of NPs for delivery offers a promising alternative toward more efficient, versatile, and species-independent gene delivery strategies.

To the best of our knowledge, the application of SWCNT-mediated plasmid DNA delivery has not yet been reported in the model monocot plant *Brachypodium distachyon*, which is widely used for functional genomics studies due to its small genome size, short life cycle, and ease of cultivation. Herein, we present the first evaluation of SWCNT-mediated plasmid DNA delivery in *B. distachyon* using both embryogenic callus tissue and seeds as target explants. Transient reporter gene activity was assessed using the *b-glucuronidase* (GUS) reporter system. By adapting and optimizing previously reported delivery parameters, the aim of the present work was to expand the applicability of SWCNT-based gene delivery approaches in monocot species.

## 2. Materials and methods

### 2.1. Plant growth conditions for in planta and callus-based experiments in Brachypodium distachyon

Mature embryos of *Brachypodium distachyon* (Bd21 line) were used as the explant source for callus induction. Seeds were surface sterilized by immersion in 70% ethanol for 5 min, followed by treatment with 53% NaOCl for 20 min and rinsing five times with sterile distilled water. Sterilized seeds were placed onto *Brachypodium* callus induction medium (BDCIM), consisting of MS basal salt medium with vitamins (4.43 g L^−1^), sucrose (30 g L^−1^), 2,4-dichlorophenoxyacetic acid (3 mg L^−1^), and plant agar (8 g L^−1^), adjusted to pH 5.8. After the embryogenic callus initiation, the seed tissues were carefully removed and the calli were transferred to fresh BCIM. Embryogenic calli were maintained in the dark at 25 °C and subcultured every 2–3 weeks (Sogutmaz Ozdemir and Budak, 2017). For the in planta experiments, *Brachypodium* seeds were surface sterilized following the previously described protocol and used directly for SWCNT-mediated plasmid DNA delivery.

### 2.2. Preparation of SWCNTs

**Figure 1:**
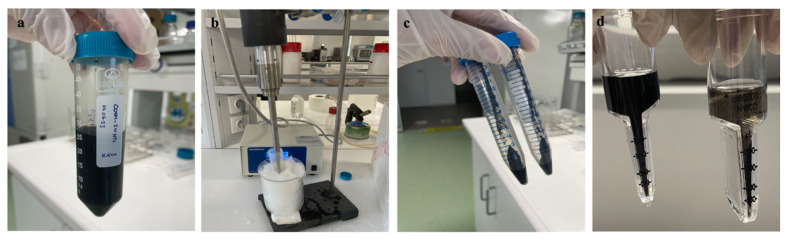
Overview of the SWCNT activation and purification process. (a) Initial COOH–SWCNT suspension, (b) probe-tip sonication performed while keeping the vial on ice to prevent heating of SWCNTs, (c) aggregated SWCNT pellet obtained after centrifugation, (d) washing steps using 100,000 MWCO filters showing homogenized suspension after bath sonication (left) and agglomeration prior to sonication (right).

SWCNTs were prepared as nanocarriers for the pCAMBIA1301 plasmid (CAMBIA, Canberra, Australia) followed by the protocol of Demirer et al. (2019b) with minor modifications. Briefly 30 mg of SWCNTs was dispersed in distilled water with a final concentration of 1 mg mL^−1^ (Figure 1a). The solution was bath-sonicated for 10 min at room temperature, followed by a probe sonication (3 mm tip, 15% amplitude) for 30 min (Figure 1b). The suspension was centrifuged at 18,000 × *g* for 1 h and the concentration of SWCNTs was determined spectrophotometrically (Figure 1c).

Then approximately 2 mg of the SWCNT solution was diluted with MES buffer solution of 100 mM final concentration. For activation of COOH–SWCNT groups, freshly prepared EDC–NHS solution (2.86 mM EDC, 18.42 mM NHS, and 100 mM MES) was added under stirring, followed by bath sonication for 15 min at room temperature. The suspension was incubated at 170 rpm for 45 min and then washed three times with 0.1X PBS using 100,000-MWCO filters. For the washing step the filters were centrifuged at 300 × *g* for 8 min at 21 °C. After a brief 1-min bath-sonication the activated COOH–SWCNTs were recovered from the filters with 1000-μL extended pipette tips, resuspended in 0.1X PBS, and bath sonicated for 15 min to ensure complete dispersion (Figure 1d).

For functionalization, activated COOH–SWCNTs were added to PEI solution (0.32 mM PEI, 0.1X PBS) and incubated overnight at 180 rpm and room temperature. The reaction mixture was washed six times with nuclease-free water using 100,000-MWCO filters and after each wash centrifuged at 1000 × *g* for 20 min at 21 °C. After a short bath-sonication for 1 min, SWCNT–PEI was recovered and resuspended to the original volume with nuclease-free water. The final suspension was bath sonicated for 15 min, followed by probe sonication (3 mm tip, 10% amplitude) for 15 min in an ice bath. After a final centrifugation at 14,000 × *g* for 1 h, the supernatant was collected, and the concentration of SWCNT–PEI was determined spectrophotometrically according to the Beer–Lambert law at 632 nm using an extinction coefficient of 0.036 mg L^−1^ cm^−1^.

### 2.3. Characterization of SWCNTs with transmission electron microscopy (TEM), atomic force microscopy (AFM), and zeta potential measurements

A JEOL JEM-2100 Plus transmission electron microscope was used for the structure and distribution analysis of the SWCNTs before and following PEI functionalization. For each sample a small aliquot was drop-cast onto a copper grid coated with carbon and air dried at room temperature. For TEM imaging of the samples, 200 kV accelerating voltage was used.

AFM was used for analysis of the surface morphology and height profiling of the SWCNTs. The sample was drop-cast on mica surface and vacuum dried before analysis (Ali et al., 2022). An XE-100 AFM system in tapping mode was used for analysis.

The zeta potential measurements of the SWCNTs were performed using a Malvern Zetasizer Nano ZS to evaluate their colloidal stability and verify the successful attachment of PEI. The dispersant was set to water and the particle properties were set to refractive index = 1.8 at 25 °C. The values of the carbon nanotubes before (COOH–SWCNT) and after functionalization (SWCNT–PEI) with PEI were measured to evaluate the efficiency of functionalization (Demirer et al., 2019a).

The concentration and dispersion quality of SWCNT suspensions were assessed via UV–Vis spectrophotometry at 632 nm wavelength. Measurements were performed with supernatant at two stages of preparation: (i) after initial dispersion to calculate the amount required for PEI functionalization, and (ii) after completion of the procedure to quantify the amount to be used for DNA loading. Concentrations were calculated according to the Beer–Lambert law with an extinction coefficient of 0.036 mg L^−1^ cm^−1^ (Demirer et al., 2019b).

### 2.4. Preparation of pCAMBIA1301 plasmid DNA

**Figure 2:**
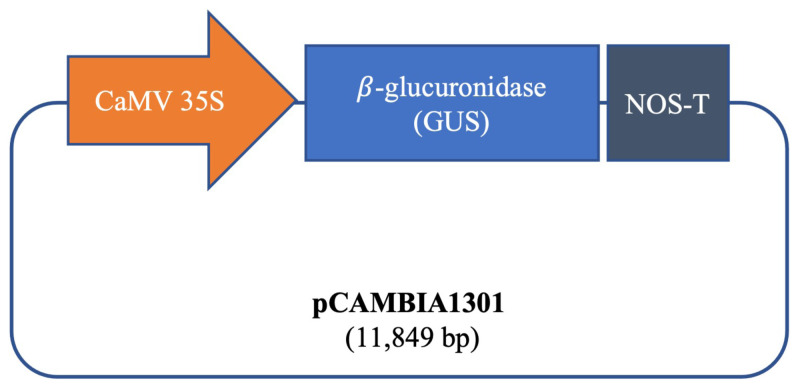
Schematic representation of the GUS gene cassette used for SWCNT-mediated transformation.

The commonly used plant binary plasmid pCAMBIA1301, encoding the GUS gene driven by cauliflower mosaic virus (CaMV) 35S promoter, was employed in the present study (Figure 2). The plasmid was transformed into *Escherichia coli* DH5a competent cells via heat shock (Sogutmaz Ozdemir and Budak, 2017). Plasmid DNA was isolated using the MN Mini Plasmid Isolation Kit following the manufacturer’s instructions, with final elution in nuclease-free distilled water, and subsequently used for SWCNT-mediated transformation experiments.

### 2.5. Loading of plasmid DNA onto SWCNTs

Calculations for the SWCNT:pDNA ratios were done according to Demirer et al. (2019b) and all reagent amounts and volumes were scaled as needed, while the SWCNT:pDNA mass ratio was kept constant. For pDNA–PEI–SWCNT complex formation, pCAMBIA1301 was first placed in an Eppendorf tube, and PEI-functionalized SWCNTs diluted in MES delivery buffer (25 mM MES, 15 mM MgCl_2_, pH 6) were then added very slowly to the pDNA to achieve the defined SWCNT:pDNA mass ratios. A total reaction volume of 200 μL was applied per callus, corresponding to 1 μg of SWCNT and 1 μg of pDNA for the 1:1 ratio, 333 ng for the 3:1 ratio, and 167 ng for the 6:1 ratio (SWCNT:pDNA, w/w). PEI–SWCNTs were used at a final concentration of 5 ng μL^−1^ in all delivery experiments. The reactions were gently mixed by pipetting 10 times and incubated for 30 min at room temperature to allow pDNA–PEI–SWCNT complex formation. No visible agglomeration was observed after incubation, consistent with stable complex formation. For seed delivery experiments, pDNA–PEI–SWCNT complexes were prepared in a total volume of 5 mL at the same final SWCNT concentration (5 ng μL^−1^), with reagent amounts scaled proportionally. Multiple SWCNT:pDNA mass ratios (1:1, 3:1, and 6:1, w/w) were initially tested in callus tissues to identify an optimal condition and, based on quantitative GUS staining analysis, the 3:1 ratio was selected for seed delivery experiments.

### 2.6. SWCNT-mediated plasmid DNA delivery of Brachypodium seeds and callus tissues

For plasmid DNA delivery into callus tissue, embryogenic calli of *Brachypodium distachyon* (Bd21) were treated by applying 200 μL of the prepared pDNA–PEI–SWCNT suspension directly onto the callus surface (approximately 500 mg fresh weight). Three treatment groups were prepared: (i) PEI–SWCNTs diluted with MES delivery buffer (only-SWCNT control), (ii) pCAMBIA1301 plasmid diluted in MES delivery buffer (only-plasmid control), and (iii) pDNA–PEI–SWCNTs complexes prepared with pCAMBIA1301 plasmid at defined SWCNT:pDNA mass ratios (1:1, 3:1, and 6:1, w/w). After application, calli were incubated at room temperature for 30 min and then transferred to BDCIM medium for recovery. Transient GUS gene expression was evaluated after 48–72 h of culture.

For the in planta delivery experiments, sterilized *Brachypodium* seeds (total of 100) were first incubated in 0.6 M mannitol solution for 3 h to promote osmotic conditioning, followed by immersion in 5 mL of the prepared pDNA–PEI–SWCNT suspension at the optimized 3:1 SWCNT:pDNA mass ratio, as determined in callus delivery experiments. PEI–SWCNTs diluted with MES delivery buffer were used as the negative control, consistent with the imbibed seed reference treatment reported by Dunbar et al. (2022). Incubation was carried out at room temperature for 5 days under gentle shaking (~100 rpm) to facilitate uniform exposure of NPs. Following incubation, the seeds were rinsed with sterile distilled water to remove residual surface associated nanomaterials and directly subjected to histochemical GUS analysis.

### 2.7. Histochemical GUS staining

Following treatment, tissues were washed multiple times with dH_2_O to remove residual surface-associated material prior to histochemical GUS staining. GUS reporter activity was visualized through histochemical staining to assess transient gene expression and estimate the efficiency of transient reporter gene expression (Jefferson et al., 1987). The β-glucuronidase enzyme, expressed from the GUS gene, catalyzes a reaction with the substrate 5-bromo-4-chloro-3-indolyl-β-D-glucoronide that results in formation of blue coloration. For GUS staining, a solution of X-gluc was prepared (0.1 M EDTA, 5 mM potassium ferricyanide, 5 mM potassium ferrocyanide, 5 mL of sodium phosphate buffer (0.1 M Na_2_HPO_4_ (61%) and 0.1 M NaH_2_PO_4_ (39%)), 0.1% Triton X-100, and 0.3 mg mL^−1^ X-gluc) and adjusted to a final volume of 10 mL with distilled water. The explants were incubated in the staining solution at 37 °C for 24 h. After the incubation, samples were examined using a Leica stereomicroscope to identify blue-stained spots indicating GUS expression.

### 2.8. Analysis of the GUS assay

GUS expression in treated *Brachypodium distachyon* tissues was quantified using ImageJ analysis according to Béziat et al. (2016) with modifications. Images were thresholded to isolate blue-stained regions corresponding to GUS activity. For each sample, the thresholded mean intensity, integrated density, and stained area were measured and normalized to the total image area to calculate the percentage of GUS-stained tissue. These quantitative values were then used to estimate relative transient reporter expression efficiency among the treatments.

### 2.9. Statistical analysis

GraphPad Prism software (version 8.4.4) was used for statistical analysis of the quantitative data. One-way analysis of variance (ANOVA) followed by Tukey’s post-hoc test was applied for comparison among multiple groups. Sample sizes (n) for each experiment are indicated in the corresponding figure legends. The data are presented as mean ± standard deviation (SD), and a p value of <0.05 was considered statistically significant.

## 3. Results

### 3.1. Characterization of SWCNTs

**Figure 3:**
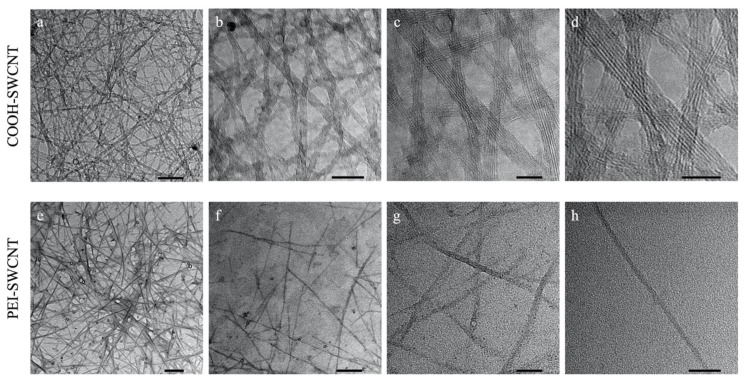
Transmission electron microscopy images of SWCNTs. (a–d) Before (COOH–SWCNT) and (e–h) after PEI functionalization (PEI–SWCNT). (a) 100 nm scale, (b) 50 nm scale, (c, d) 20 nm scale, (e, f) 200 nm scale, (g, h) 50 nm scale.

TEM was used to examine the morphological characteristics of the synthesized SWCNTs before and after PEI functionalization, as well as their dispersion quality. The TEM images showed that the synthesized SWCNTs were well dispersed and exhibited nanoscale dimensions. The pristine SWCNTs appeared to be slightly elongated and formed in bundles with diameters ranging from 10 to 20 nm. Initially, carboxylated SWCNTs (COOH-SWCNT) dispersed and sonicated in water appeared as aggregated bundles, which was expected due to their hydrophobic nature (Figures 3a–3d). After the sonication and PEI functionalization, TEM images clearly showed a transition towards abundant individually dispersed, needle-like nanotube structures (Figures 3e–3h). The observation of this needle-like structure is correlated to the functional mechanism of SWCNTs, as it is consistent with their reported ability to pass through the plant cell wall during cargo delivery. This morphological change confirms the efficiency of dispersion and functionalization processes.

**Figure 4:**
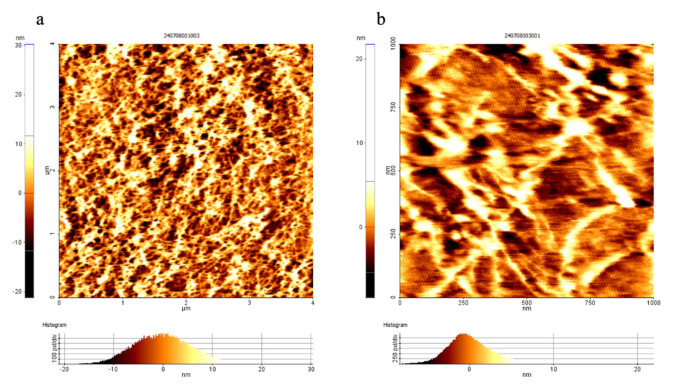
Atomic force microscopy images of COOH-functionalized SWCNTs. (a) scan size 4 × 4 μm, and (b) scan size 1 × 1 μm.

The surface morphology of COOH–SWCNTs was characterized using the tapping mode of AFM (Figures 4a and 4b). The resulting AFM images showed a characteristic elongated network structure, in line with the results from the TEM. It was observed that the SWCNTs were well dispersed, maintaining their elongated nanotube structures. The observed height profile of below 10 nm was also consistent with expectations.

### 3.2. Zeta potential analysis of nanomaterial–DNA complexes

**Figure 5:**
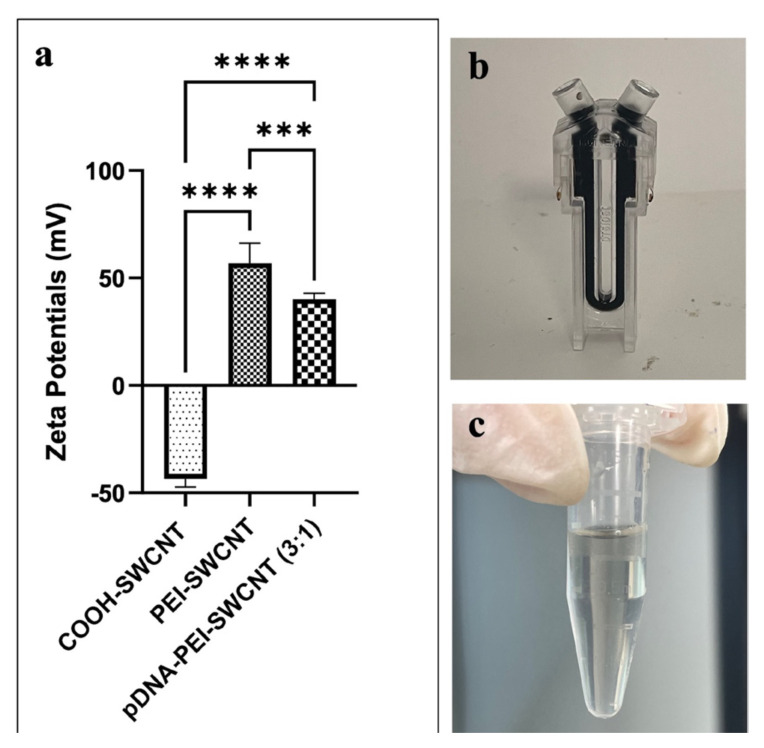
Characterization of SWCNT dispersion stability and surface charge modification. (a) Zeta potential measurements of COOH–SWCNTs, PEI–SWCNTs, and pDNA–PEI–SWCNT complexes (3:1) measured by DLS (statistical analysis was performed using one-way ANOVA (n = 6 per group). ***p < 0.001, ****p < 0.0001. Data represent mean ± SD, (b) uniformly dispersed PEI–SWCNT solution in a Zetasizer cuvette prior to measurement of DLS, (c) the expected SWCNT stability behavior after pDNA loading.

Electrostatic grafting was used to load plasmid DNA on SWCNTs by creating a covalent conjugation between the cationic polymer PEI and COOH–SWCNTs. Zeta potential measurements were used for the confirmation of successful PEI attachment, as a positive surface charge indicated stable colloidal dispersion suitable for biological applications, in this case providing favorable conditions for DNA binding. As shown in Figure 5a, the initial negative zeta potential of −43.45 ± 8.13 mV, due to the COOH groups on SWCNTs, shifts toward +56.95 ± 6.14 mV following PEI functionalization, which is within the range reported in previous studies (Demirer et al., 2019a). The positive shift confirmed the successful polymer binding and established favorable electrostatic conditions for DNA binding, which is critical for nanomaterial-based delivery systems. A subsequent decrease in zeta potential after incubation with plasmid DNA (40.20 ± 6.14 mV) further confirmed effective plasmid attachment to the PEI–SWCNT complex. The differences in zeta potential among all three groups were statistically significant. Throughout the preparation, aggregation was carefully monitored to ensure stable dispersion. All solutions were sonicated prior to zeta potential analysis. For consistency, the preparation step was repeated if any aggregation was observed. As shown in Figures 5b and 5c, the absence of visible aggregation confirms the stability of the PEI-functionalized SWCNT suspensions.

### 3.3. Transient GUS expression in callus tissues treated with pDNA–PEI–SWCNT complexes

**Figure 6:**
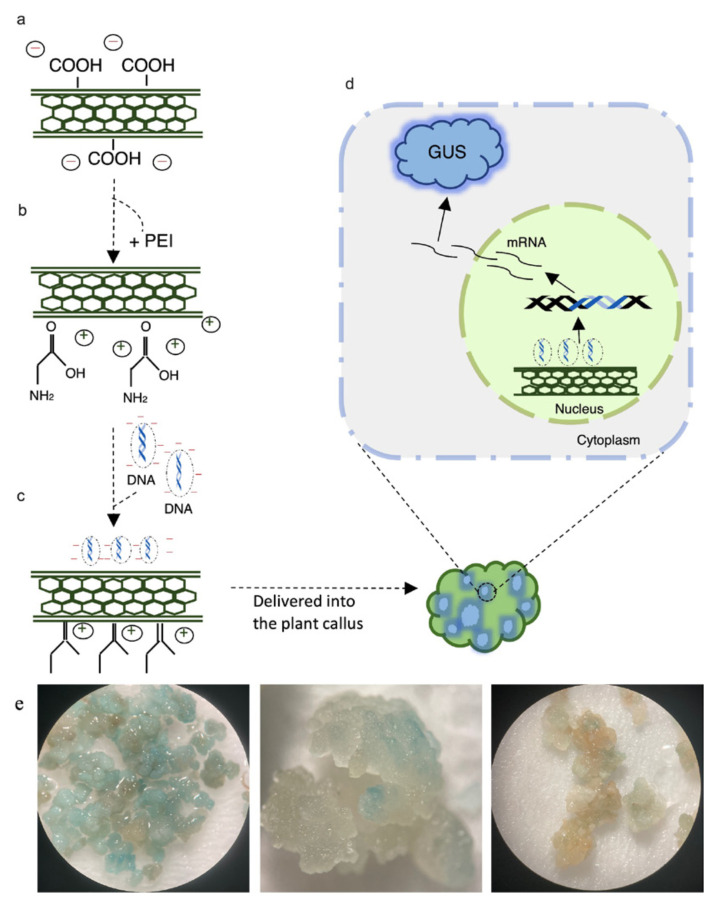
Schematic representation of the SWCNT-mediated plasmid DNA delivery and GUS expression in *Brachypodium* tissues. (a) Negatively charged carboxylated SWCNTs, (b) PEI-functionalized positively charged SWCNTs, (c) complex formation with pDNA via electrostatic interaction, (d) delivery into callus tissues, and subsequent expression of introduced genes, (e) transient expression of the GUS reporter gene in *Brachypodium* callus tissues, observed under a stereomicroscope at 8×, 16×, and 8× magnifications (from left to right, respectively). The third callus image on the right represents the only-SWCNT treated control callus.

A schematic overview was prepared to illustrate the process of SWCNT-mediated gene delivery into callus tissues (Figure 6). Carboxylated SWCNTs were functionalized with PEI for generation of positively charged NPs, which enables binding of plasmid DNA via electrostatic interactions (Figures 6a–6c). The established pDNA–PEI–SWCNT delivery protocol was applied to *Brachypodium* callus to assess transient expression of the GUS reporter gene on pCAMBIA1301 (Figure 6d). Following incubation for 2–3 days to allow gene expression and protein accumulation, callus tissues were subjected to histochemical GUS staining. Representative stereomicroscopy images show clear blue coloration in treated calli following histochemical GUS staining, indicating successful delivery and transient expression of the reporter gene (Figure 6e).

**Figure 7:**
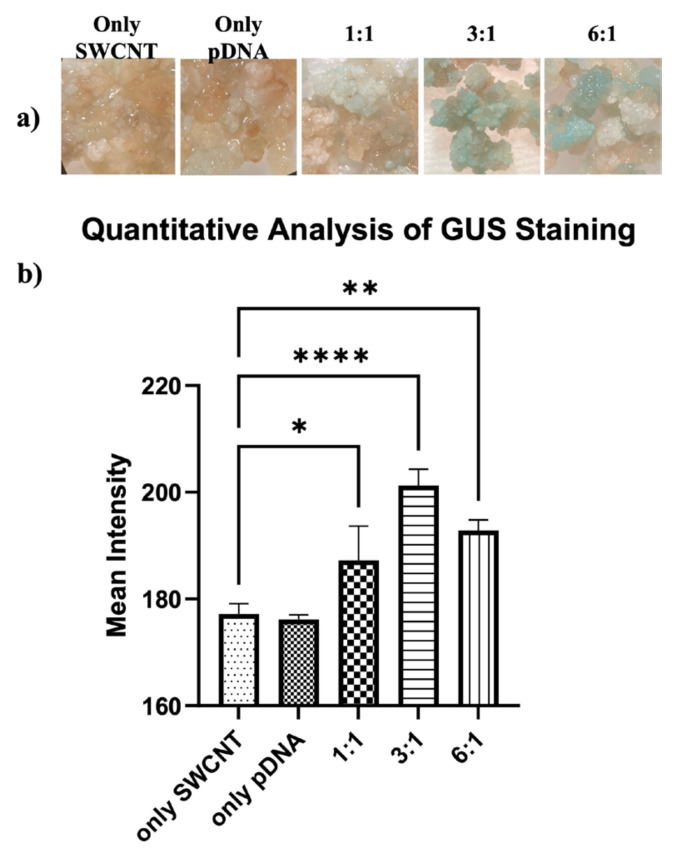
Transient GUS expression in *Brachypodium distachyon* callus tissues following SWCNT-mediated pCAMBIA1301 delivery. (a) Representative stereomicroscope images of calli treated with only-SWCNT in MES buffer, only-pDNA in MES buffer, and pDNA-PEI-SWCNT complexes at 1:1, 3:1, and 6:1 SWCNT:pDNA mass ratios, shown from left to right, respectively (10× magnification), (b) quantification of GUS staining intensity of calli treated with only-SWCNT in MES buffer (only-SWCNT), only-pDNA in MES buffer only-pDNA, and pDNA–PEI–SWCNT complexes at 1:1, 3:1, and 6:1 ratios, illustrating relative levels of transient reporter activity. Data represent mean ± SD of three biological replicates (n = 3). Statistical significance was determined by one-way ANOVA followed by Tukey’s multiple comparison test (*p < 0.05, **p < 0.01, ****p < 0.0001).

GUS staining was quantified using ImageJ by measuring mean intensity values, which reflect the average signal strength per unit area. Following treatment with pDNA–PEI–SWCNT complexes, callus tissues exhibited clear and distinct GUS staining, whereas control samples treated with only-pDNA or only-SWCNT showed no visible staining (Figure 7a). In the callus experiments, the only-pDNA treatment was included as an additional negative control, consistent with previous SWCNT-mediated delivery studies, to confirm that reporter expression does not occur in the absence of SWCNTs. Any apparent signal in control samples likely reflected nonspecific background staining rather than true enzymatic activity. Quantitative analysis showed no significant difference between the only-SWCNT and only p-DNA controls (p = 0.9964), indicating similar low background staining in both conditions.

Quantitative analysis of GUS staining showed that all pDNA–PEI–SWCNT treatments resulted in significantly higher signals compared to the only-SWCNT control, which confirms that SWCNTs are required for efficient plasmid delivery into callus tissues (Figure 7b). Among the SWCNT:pDNA mass ratios tested, both the 3:1 and 6:1 ratios resulted in significantly higher staining intensity compared to the only-SWCNT control (p < 0.0001 and p < 0.01, respectively), while the 1:1 ratio showed weaker expression (p < 0.05). No statistically significant difference was detected between the 3:1 and 6:1 treatments. The 3:1 ratio exhibited the highest mean staining intensity; hence it was selected for subsequent seed delivery experiments.

### 3.4. Transient GUS expression in seeds treated with pDNA–SWCNT complexes

**Figure 8:**
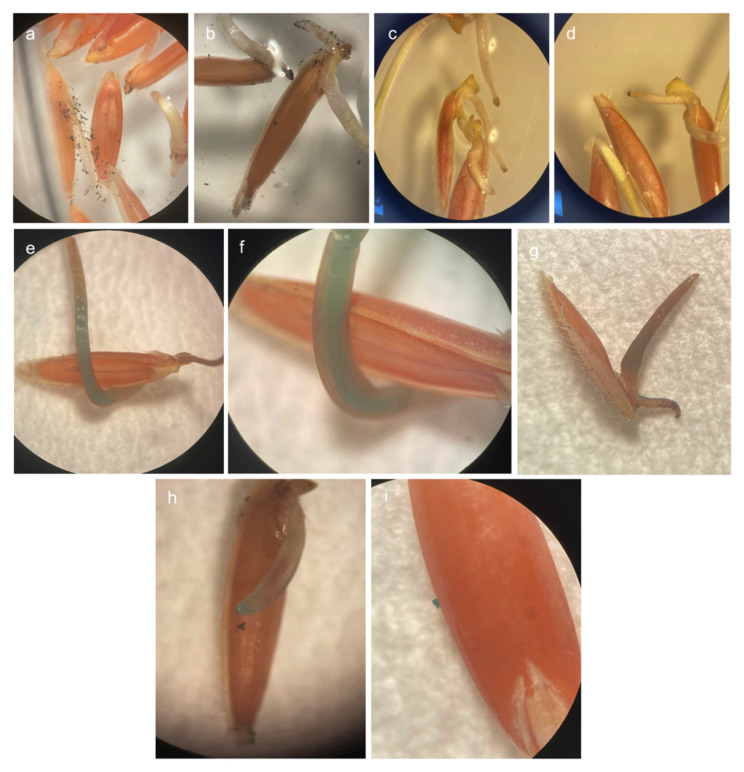
SWCNT-mediated pCAMBIA1301 delivery in *Brachypodium distachyon* seeds. (a, b) Seeds incubated for 5 days in pDNA–PEI–SWCNT solution prior to GUS staining (8× and 10× magnification, respectively), (c, d) seeds treated with only-SWCNT (negative control) subjected to GUS staining, showing no detectable GUS activity (8× magnification), (e–i) GUS positive seeds exhibiting blue coloration, indicating successful transient expression (stereomicroscopy images at 8×, 32×, 10×, 20×, and 32× magnification, respectively).

Quantitative analysis of the GUS staining of callus tissues revealed that the 3:1 SWCNT:pDNA (w/w) ratio resulted in significantly higher reporter expression compared with the other ratios tested. Therefore, the 3:1 ratio was selected for seed-based delivery experiments. Seeds were incubated with pDNA–SWCNT solution in 3:1 ratio (5 ng/μL SWCNT) for 5 days, followed by thorough washing with sterile distilled water to remove residual and loosely associated nanomaterials (Figures 8a and 8b). Then they were directly subjected to GUS reporter activity. The seeds were shaken at 100 rpm to ensure homogeneous exposure to NPs. Under the tested conditions, no obvious morphological abnormalities were observed. No vacuum infiltration was applied at this stage, which is usually recommended to assess whether SWCNTs could facilitate plasmid DNA delivery into seed tissues without additional physical assistance. In total, 4% of the treated seeds exhibited detectable GUS staining across biological replicates, while no reporter activity was observed in seeds treated with only-SWCNT control solution (Figures 8c and 8d). Blue staining was detected in the embryo and scutellum as well as on the outer pericarp surface of the seeds (Figures 8e–8i). GUS expression was observed in the developing hypocotyl tissue of *Brachypodium* seeds, suggesting localized reporter activity following SWCNT-mediated plasmid DNA delivery. These results demonstrate that SWCNT-mediated delivery can enable transient reporter activity in seed tissues of *Brachypodium distachyon*, supporting the potential of this approach for transient expression-based applications (Demirer et al., 2019a; Dunbar et al., 2022). Further optimization may improve delivery outcomes depending on species and explant source.

## 4. Discussion

In the present study, we demonstrated for the first time the preparation, characterization, and successful use of SWCNTs as plasmid carriers in *Brachypodium distachyon* calli and seeds. TEM imaging, zeta potential analysis, and UV–Vis spectrophotometry confirmed the synthesis and stability of pDNA–PEI–SWCNT complexes, while GUS reporter expression validated their ability to mediate transient gene delivery. This establishes a new platform for exploring NP-mediated delivery and transient gene expression in monocots.

Efficient NP synthesis and functionalization play a major role in the efficiency for SWCNT-mediated transformations. SWCNTs are hydrophobic and prone to aggregation, which limits their cargo loading and penetration efficiency. COOH–SWCNTs were activated using the EDC–NHS duo to enhance the reactivity of surface carboxyl groups. EDC forms a highly reactive O-acylisourea intermediate, which is subsequently stabilized by NHS to produce an active ester, enabling efficient covalent bonding with amine-containing ligands such as PEI. Due to its stability in aqueous environments and higher efficiency in ligand attachment, EDC–NHS is preferred to other methods such as thermal activation (Demirer et al., 2019a). In the present study, electrografting was used to create a positively charged environment to facilitate binding of our cargo, negatively charged plasmid DNA. For this purpose, PEI, a cationic and amine rich polymer, was employed to functionalize the carboxylated SWCNTs to produce stable and functionalized nanomaterials. Upon functionalization, PEI’s hydrophilic nature reduces this tendency, resulting in better dispersion (Parveen et al., 2024). Our results indicated functionalization, as shown by the positive shift in zeta potential.

Previous studies suggest that surface charge, particle size, and functionalization collectively influence the ability of SWCNTs to pass through the plant cell wall (Wong et al., 2016; Demirer at al., 2019a, 2019b; González-Grandío et al., 2021; Liu et al., 2023). The zeta potential shift served as a key indicator of successful functionalization; measurements were taken both during preparation and of the final product. Initially, the COOH–SWCNTs exhibited a negative surface charge of approximately −43 mV, caused by the carboxyl groups attached to the SWCNTs. Following PEI functionalization, the zeta potential shifted to +56 mV, which is within the expected range (Demirer et al., 2019b). Although previous reports demonstrated successful pDNA delivery at approximately +34.03 mV, no expression was detected in our trials at similar values (Pinyokham et al., 2022). This suggests that surface zeta potential has a critical role in efficient delivery of large plasmids. Similar findings have been reported when SWCNTs were functionalized with positively charged polymers such as PEG or DSPE, which enhances DNA loading of carbon nanotubes (Yang et al., 2020). The results of zeta potential measurements confirmed that the SWCNTs are suitable for stable cargo loading, an essential requirement for efficient delivery into plant cells. Moreover, UV–Vis spectrophotometry enabled us to confirm the homogeneity of dispersion and provided the quantification of SWCNT and PEI–SWCNT suspensions, which are key parameters for reproducible delivery outcomes.

A major bottleneck in plant transformation is the difficulty of overcoming the physical plant cell wall barrier, which has a size exclusion limit of 5 to 20 nm (Carpita and Gibeaut, 1993). TEM and AFM analyses confirmed that the synthesized SWCNTs were within this nanoscale range, supporting their potential to penetrate through the cell wall due to their elongated, needle-like morphology. This structural feature provides a significant advantage over conventional transformation systems, such as *Agrobacterium*-mediated transformation or biolistic approaches, which are constrained by cell wall permeability limitations. In *Agrobacterium*-mediated gene transfer, transfer of T-DNA across the cell wall is genotype-dependent, whereas, in the biolistic method, the penetration of DNA-coated particles through the cell wall can cause physical damage, resulting in low transformation efficiency.

The binary vector pCAMBIA1301 was successfully delivered into *Brachypodium* callus tissues and seeds using SWCNTs, as indicated by transient GUS reporter activity in both explant types. Of the 1:1, 3:1, and 6:1 SWCNT:pDNA ratios tested, the 3:1 ratio produced the highest GUS staining intensity, suggesting an optimal balance between efficient DNA loading and complex stability. This observation is consistent with previous reports highlighting the importance of the SWCNT to cargo ratio for delivery efficiency (Demirer et al., 2019b; Dunbar et al., 2022). For SWCNT-based plasmid delivery in the seed experiments, localized GUS activity was observed in *Brachypodium* seed-associated tissues, indicating successful plasmid DNA delivery, similar to observations in a previous study on rice in which SWCNTs facilitated transient GUS expression in embryo and seed tissues (Dunbar et al., 2022). Although the plasmid delivery experiments in seeds were conducted using the 3:1 SWCNT:pDNA ratio optimized in callus tissues, differences in uptake mechanisms and exposure conditions between callus and seed tissues may influence delivery efficiency; therefore, future work should explore a wider range of SWCNT:pDNA ratios and conditions for further optimization.

The plasmid used in the present study (~12 kb) represents one of the largest constructs successfully delivered into plant tissue using SWCNTs. Plasmid DNA (pDNA) is known to be more stable within plant cells than linear DNA due to the absence of free ends susceptible to nucleolytic degradation (Zhuang et al., 2024). In addition, PEI-functionalized SWCNTs are well suited for complexing and delivering circular plasmids due to their elongated morphology and positive surface charge. Accordingly, most SWCNT-mediated gene delivery studies have focused on pDNA as their cargo (Demirer et al., 2019a; Ali et al., 2022; Dunbar et al., 2022; Pinyokham et al., 2022; Santana et al., 2022). Consistent with this, a recent study comparing different NPs and cargos showed that pDNA was more efficient than linear DNA for SWCNT-mediated delivery (Şener et al., 2026). However, it has been suggested that although SWCNTs have a high surface area for binding, condensed packing can limit access to the plant’s transcriptional machinery, thereby reducing transgene expression (Ali et al., 2022). Our findings suggest that large plasmids can be delivered via SWCNT-mediated gene delivery within the size range tested; however, optimization of the SWCNT to cargo ratio remains critical.

Once the plasmid is effectively delivered into the plant cell, the transgene expression is determined by the promoter driving the reporter gene. In the present study, the CaMV 35S promoter was employed for constitutive expression. However, for seed-based delivery approaches, the use of tissue-specific (e.g., egg-cell specific) or inducible promoters could potentially enhance transgene expression in target tissue while minimizing negative effects on overall plant development (Biłas et al., 2016; Zhou et al., 2020).

In our seed-based assays, which represent an in planta style delivery approach, a 5-day incubation period with pDNA–SWCNT complex was required to achieve detectable GUS expression, whereas shorter incubation times (1–3 days) produced no visible reporter activity in our experiments. This timing likely corresponds to early germination stages when seed coats rupture and metabolic activity increases, which may have facilitated transient gene expression. This observation is consistent with earlier reports suggesting that 3–6 days of posttreatment growth is necessary for transient expression. Additionally, the pretreatment of seeds with mannitol prior to SWCNT exposure was also found to be effective, consistent with findings from rice embryos (Dunbar et al., 2022).

The seeds were submerged in a 5 ng μL^−1^ SWCNT solution and showed no visible signs of necrosis or growth inhibition during germination, consistent with previous reports that indicate SWCNT exposure at concentrations below 10 mg L^−1^ does not cause detectable cytotoxicity or observable tissue damage in plants (Demirer et al., 2019a; Kwak et al., 2019). Previous reports also show that SWCNTs can penetrate seed coats and promote germination in several species, including tomato, barley, soybean, and corn (Khodakovskaya et al., 2009; Lahiani et al., 2013; Pandey et al., 2018; Ali et al., 2022).

GUS is commonly recommended for first-stage optimization trials in monocot delivery and transformation systems (Hayta et al., 2021). The histochemical GUS assay, which remains a widely used and reliable reporter system in plant transformation, provided functional evidence that the pDNA–SWCNT complexes were able to mediate gene transfer resulting in transient reporter activity in *Brachypodium* callus tissues. This transient expression approach in callus could offer a rapid platform for analyzing promoter activity or screening constructs, representing a practical and time-efficient alternative to conventional transformation methods.

In the present study, transient GUS expression was observed, which is consistent with previous reports suggesting that PEI–SWCNTs deliver plasmid DNA across the cell wall and plasma membrane into the cytoplasm and nucleus via electrostatic interactions (Demirer et al., 2019a). Unlike *Agrobacterium*-mediated transformation or biolistics, this mechanism does not induce DNA breaks or provide the recombination machinery required for stable integration, causing the delivered plasmid to remain episomal and is gradually diluted or degraded during cell division, which leads to temporary expression and minimizes biosafety concerns (Demirer et al., 2019a). Importantly, the successful delivery of a large plasmid in the present study, comparable in size to typical CRISPR/Cas constructs, highlights the potential of SWCNT-mediated transformation as an efficient, transgene-free platform for plant genome editing in *Brachypodium*.

The current study presents the first demonstration of SWCNT-mediated DNA delivery into the model plant *Brachypodium distachyon*, highlighting its novelty and potential as a transient gene delivery platform in monocot plants. Taken together, our findings demonstrate the promising potential of SWCNT-mediated delivery for achieving transient gene expression and enabling preliminary seed-stage (in planta) delivery applications. While further optimization is required to improve efficiency, the present work establishes a foundation for developing NP-based plant transformation strategies that address the key limitations of *Agrobacterium*-mediated and biolistic methods. Overall, these results support the future development of transient gene expression and genome editing strategies in cereal crops.

## Figures and Tables

**Table:** Comparison of common plant transformation methods (adapted from Keshavareddy et al., 2018).

Delivery Method	Target tissue	Cargo type	Advantages	Limitations
*Agrobacterium*-mediated delivery	Mature plants, immature tissue (calli, meristems, embryos)	Limited to DNA, large cargo size	Suitable for large sizes of DNA, high efficiency, stable transformation, no specialized equipment	Can lead to apoptosis/necrosis, random genomic integration, requires tissue culture, limited host range (monocots)
Biolistic particle mediated delivery	Calli, embryos, leaves	DNA, siRNA, miRNA, ribonucleoproteins (RNPs), large cargo size	Suitable for large sizes of DNA, rapid protocol, applicable to many species	Physical damage to target tissue and cargo, low efficiency, random integration, multiple-copy transgene number
PEG-mediated delivery	Protoplast	Nucleic acids (DNA, siRNA, miRNA)	Applicable to different plants, no specialized equipment, cost effective, transient	Requires protoplast isolation, poor regeneration capacity
